# Biomarker enrichment medium: A defined medium for metabolomic analysis of microbial pathogens

**DOI:** 10.3389/fmicb.2022.957158

**Published:** 2022-07-22

**Authors:** Maryam Mapar, Thomas Rydzak, Ryan A. Groves, Ian A. Lewis

**Affiliations:** Department of Biological Science, University of Calgary, Calgary, AB, Canada

**Keywords:** biomarker enrichment medium, Mueller Hinton, metabolomics, LC-MS, biomarkers

## Abstract

Microbes have diverse metabolic capabilities and differences in these phenotypes are critical for differentiating strains, species, and broader taxa of microorganisms. Recent advances in liquid chromatography-mass spectrometry (LC-MS) allow researchers to track the complex combinations of molecules that are taken up by each cell type and to quantify the rates that individual metabolites enter or exit the cells. This metabolomics-based approach allows complex metabolic phenotypes to be captured in a single assay, enables computational models of microbial metabolism to be constructed, and can serve as a diagnostic approach for clinical microbiology. Unfortunately, metabolic phenotypes are directly affected by the molecular composition of the culture medium and many traditional media are subject to molecular-level heterogeneity. Herein, we show that commercially sourced Mueller Hinton (MH) medium, a Clinical and Laboratory Standards Institute (CLSI) approved medium for clinical microbiology, has significant lot-to-lot and supplier-to-supplier variability in the concentrations of individual nutrients. We show that this variability does not affect microbial growth rates but does affect the metabolic phenotypes observed *in vitro*—including metabolic phenotypes that distinguish six common pathogens. To address this, we used a combination of isotope-labeling, substrate exclusion, and nutritional supplementation experiments using Roswell Park Memorial Institute (RPMI) medium to identify the specific nutrients used by the microbes to produce diagnostic biomarkers, and to formulate a Biomarker Enrichment Medium (BEM) as an alternative to complex undefined media for metabolomics research, clinical diagnostics, antibiotic susceptibility testing, and other applications where the analysis of stable microbial metabolic phenotypes is important.

## Introduction

Microbial phenotypes are dictated not only by their inherent metabolic capabilities (dictated by genes and protein expression), but also by their nutritional environment. The effect of medium composition on biological phenotypes [i.e., growth rates and biomass (Mah et al., 1967; Letort and Juillard, 2001; Zhang et al., 2009; Texeira et al., 2015; Machado et al., 2019; Sanchez-Rosario and Johnson, 2021)], and bioproduct yields [i.e., exopolysaccharides, proteins, biofuels (DeBell, 1979; Grobben et al., 1998; Torino et al., 2005; Islam et al., 2013; Khan et al., 2013; Verbeke et al., 2017)] is well-documented. Nutritional availability can not only affect cell growth, but can direct metabolism toward the production of certain bioproducts.

Significant biological insights can be gained by quantifying the rates at which molecules are consumed and secreted by cells (Teusink et al., 2006). These boundary fluxes can be used to systematically screen isolates for auxotrophies (Levering et al., 2016; Basile et al., 2020), construct computational networks to understand cellular metabolism and nutrient-product relationships (Steuer, 2007; Copeland et al., 2012), optimize bioproduct yields and rates (Laiglecia et al., 2013; Joshi et al., 2017), and differentiate species and antimicrobial susceptibilities for diagnostics (Rydzak et al., 2022). With the evolution of high-resolution liquid chromatography-mass spectrometry (LC-MS), there has been an increasing emphasis on capturing a broad range of molecular targets for boundary flux analysis. These new approaches now capture a large transect in central carbon metabolism, allowing us to better understand intracellular metabolomic phenotypes.

One of the challenges in quantifying metabolic boundary fluxes is that microbial phenotypes are sensitive to medium composition. These minor variations in nutrients can dramatically affect the metabolic phenotype observed *in vitro* (Sánchez et al., 2000; McGillicuddy et al., 2018). This is particularly problematic when microbial phenotypes are being used for diagnostic purposes that require reproduceable quantification of limited biomarker sets. This reproducibility can be impacted when using complex medium. While the production of complex medium is well-standardized by suppliers, it does contain many chemical species in unknown proportions derived from biological sources (i.e., yeast or beef extract, casein hydrolysate, etc.). Although the bulk composition of these media with respect to lipids, proteins, and nutrients is stable enough to maintain consistent growth, the specific concentrations of nutrients present in these media are not well-controlled.

Herein, we show that commercially sourced Mueller Hinton (MH) medium, a CLSI approved medium for clinical microbiology, has significant lot-to-lot and supplier-to-supplier variability in the concentrations of individual nutrients. To demonstrate that this variability directly translates into instability in metabolic phenotypes, we employed a previously published metabolic preference assay (MPA) that is capable of differentiating common bloodstream pathogens and testing their antibiotic susceptibility *in vitro* following a short 4 h incubation (Rydzak et al., 2022). Specifically, as little as six biomarkers including arabitol, urocanate, succinate, xanthine, mevalonate and N^1^,N^12^-diacetylspermine can differentiate six common bloodstream pathogens (*Candida albicans, Klebsiella pneumoniae, Escherichia coli, Pseudomonas aeruginosa, Staphylococcus aureus*, and *Enterococcus faecalis*). While variability in metabolomic phenotypes on different lots of media was encountered during the rapid clinical assay for bloodstream infections (BSI), this is a generalized problem in any metabolomic assay that seeks to collect metabolomic phenotypes using a boundary flux approach. To address this, we developed a defined, complex medium, derived from nutrient-rich Roswell Park Memorial Institute (RPMI) medium, that supports the growth of pathogens, restores the metabolic biomarkers used to differentiate six common bloodstream infection species, and allows for antibiotic susceptibility testing (AST). Using a combination of isotope-labeling, substrate exclusion, and nutritional supplementation experiments, we identified the specific nutrients used by the microbes in the production of these biomarkers, as well as the minimal complement of nutrients needed to support their growth *in vitro*. We propose this newly formulated Biomarker Enrichment Medium (BEM) as an alternative to complex undefined media for metabolomics research, clinical diagnostics, and other applications where the analysis of stable microbial metabolic phenotypes is important.

## Results

### Batches of Mueller Hinton medium differ in nutritional composition

**Figure 1:**
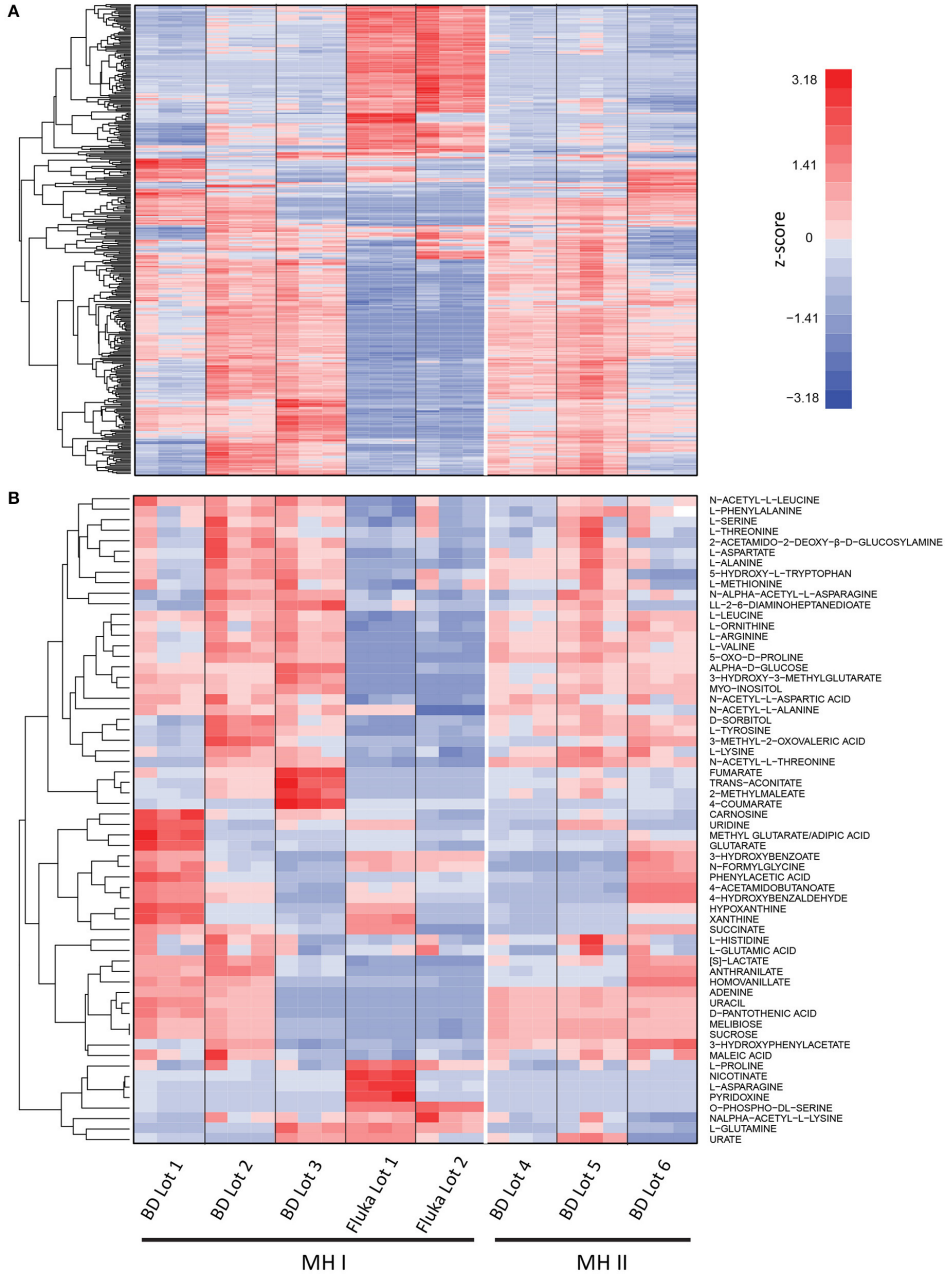
Mass spectrometry analysis of different lots and suppliers of Mueller Hinton (MH I) and cation adjusted Mueller Hinton (MH II) medium. **(A)** Untargeted analysis identified significant differences (*p* < 1 × 10^−5^) in 359 features were observed in at least one lot of medium (*n* = 3). **(B)** Targeted UHPLC-MS analysis identified variability in concentrations of many common microbial nutrients, including various amino acids, nucleotides, and sugars. Signal intensities are shown as z-scores (i.e., mean centered, variance stabilized signal intensities). See Source Data File 1.

To assess variability in MH medium, non-cation adjusted MH medium (MH I) and cation adjusted MH medium (MH II), from two different suppliers (BD and Fluka Analytical; See Materials and Methods), was analyzed using untargeted ultra high-pressure liquid chromatography-mass spectrometry (UHPLC-MS). One way ANOVA identified variability in 359 features when using the stringent cut-offs (*p* < 1 × 10^−5^) between the 8 batches of MH medium tested (Figure 1A; Source Data File 1). Even lot-to-lot variability of both MH I and MH II was observed from the same suppliers. Targeted UHPLC-MS analysis identified variability in concentrations of many common microbial nutrients, including various amino acids, nucleotides, and sugars (Figure 1B; Source Data File 1). This lot-to-lot nutritional variability was unsurprising given that MH medium is composed of undefined components including beef extract and casein hydrolysate.

### Differences in metabolomic phenotypes are observed on different batches of medium

**Figure 2:**
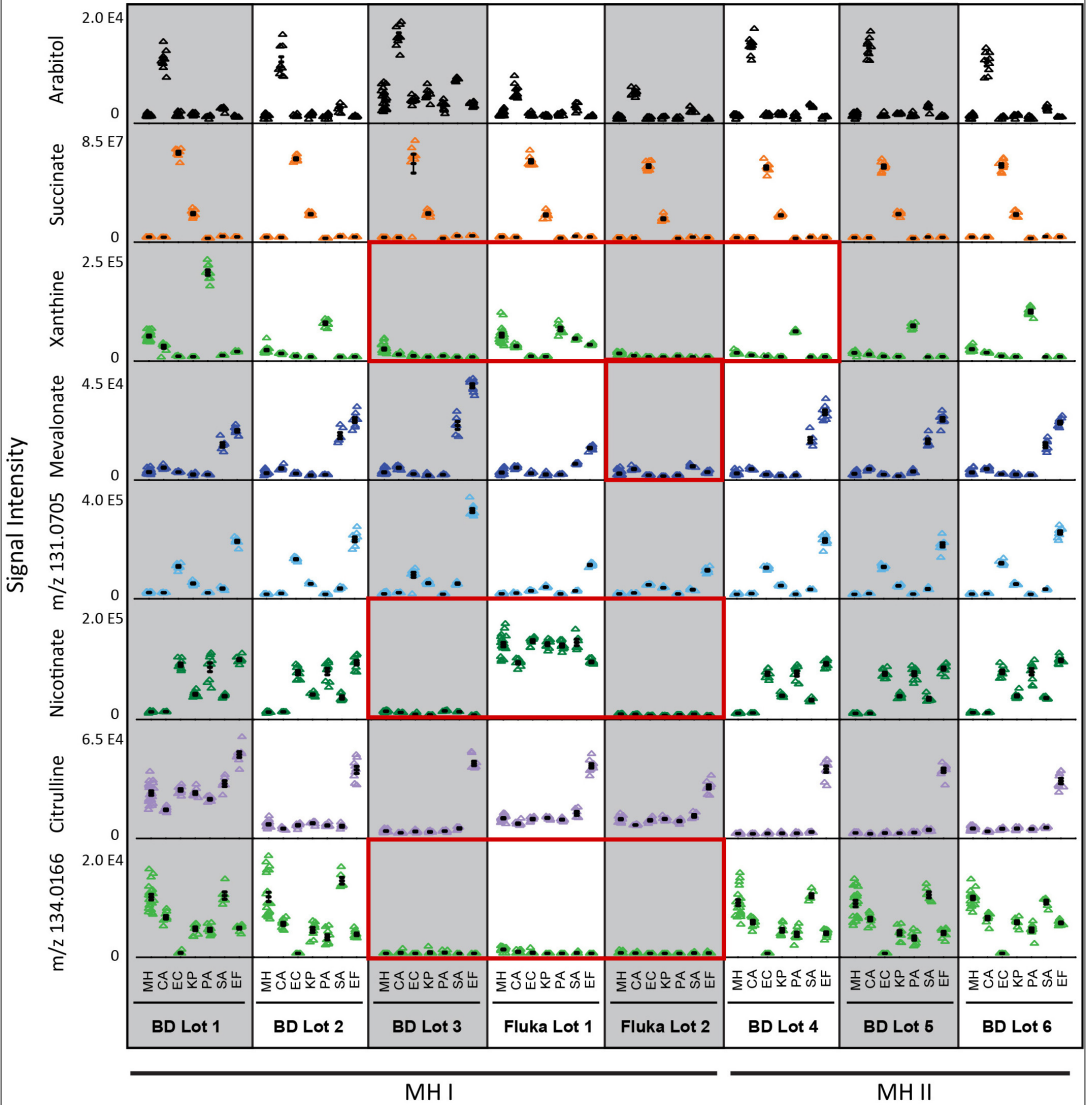
Differential biomarker production used to differentiate the six common species responsible for bloodstream infections incubated in different batches of MH medium. Three of the eight batches studies (BD Lot 3, and Fluka Lots 1 and 2) showed greatest inconsistencies with regards to species-specific production of xanthine, nicotinate, and mevalonate (Fluka Lot 2 only), and initial medium levels of unassigned marker with *m/z* 134.0166 (Source Data File 2). Key biomarkers that are affected by certain lots of media have been highlighted in red boxes. MH, Mueller Hinton medium; CA*, Candida albicans*; KP*, Klebsiella pneumoniae*; EC*, Escherichia coli*; PA*, Pseudomonas aeruginosa*; SA*, Staphylococcus aureus*; EF*, Enterococcus faecalis*. Data represents *n* = 9 biological replicates.

To determine if this nutritional variability affects microbial metabolic boundary fluxes, six prevalent pathogens responsible for BSIs (*Candida albicans*, CA; *Klebsiella pneumoniae*, KP; *Escherichia coli*, EC; *Pseudomonas aeruginosa*, PA; *Staphylococcus aureus*, SA; and *Enterococcus faecalis*, EF) were incubated for 4 h on each lot of MH medium and previously identified biomarkers used to differentiate these species were measured by targeted UHPLC-MS (Figure 2, Source Data File 2) (Rydzak et al., 2022). In some cases, the production of biomarkers in inoculated vs. non-inoculated medium was sufficient to differentiate these pathogens in all batches of medium. These include arabitol production by CA, succinate production by EC and KP. However, batch-dependent variability was also observed in many cases where species-specific biomarker production did not exceed that of MH non-inoculated controls. This was true for (i) mevalonate production in Fluka Lot 2 for SA and EF, (ii) xanthine production in BD Lot 3 and Fluka Lots 1 and 2 for PA, and (iii) nicotinate production in BD Lot 3 and Fluka Lots 1 and 2 for EC, KP, PA, SA, and EF. Notably, non-inoculated MH control nicotinate levels were much higher in Fluka Lot 1 than in all other lots. This was also true for citrulline in BD Lot 1. Lastly, the concentration of the unassigned marker with *m/z* 134.0166 was negligible in BD Lot 3 and Fluka Lots 1 and 2, and thus consumption by CA, EC, KP, PA, and EF was not observed in these lots. Given that all samples were incubated on the same day and analyzed on the MS concurrently, we can rule out that this variance is due to differences in processing or MS response factors. These discrepancies in lot-to-lot biomarker changes demonstrate the need for the use of a more standardized medium to ensure consistency when performing metabolomic studies.

### Biomarker production levels are lower on defined RPMI vs. MH

**Figure 3:**
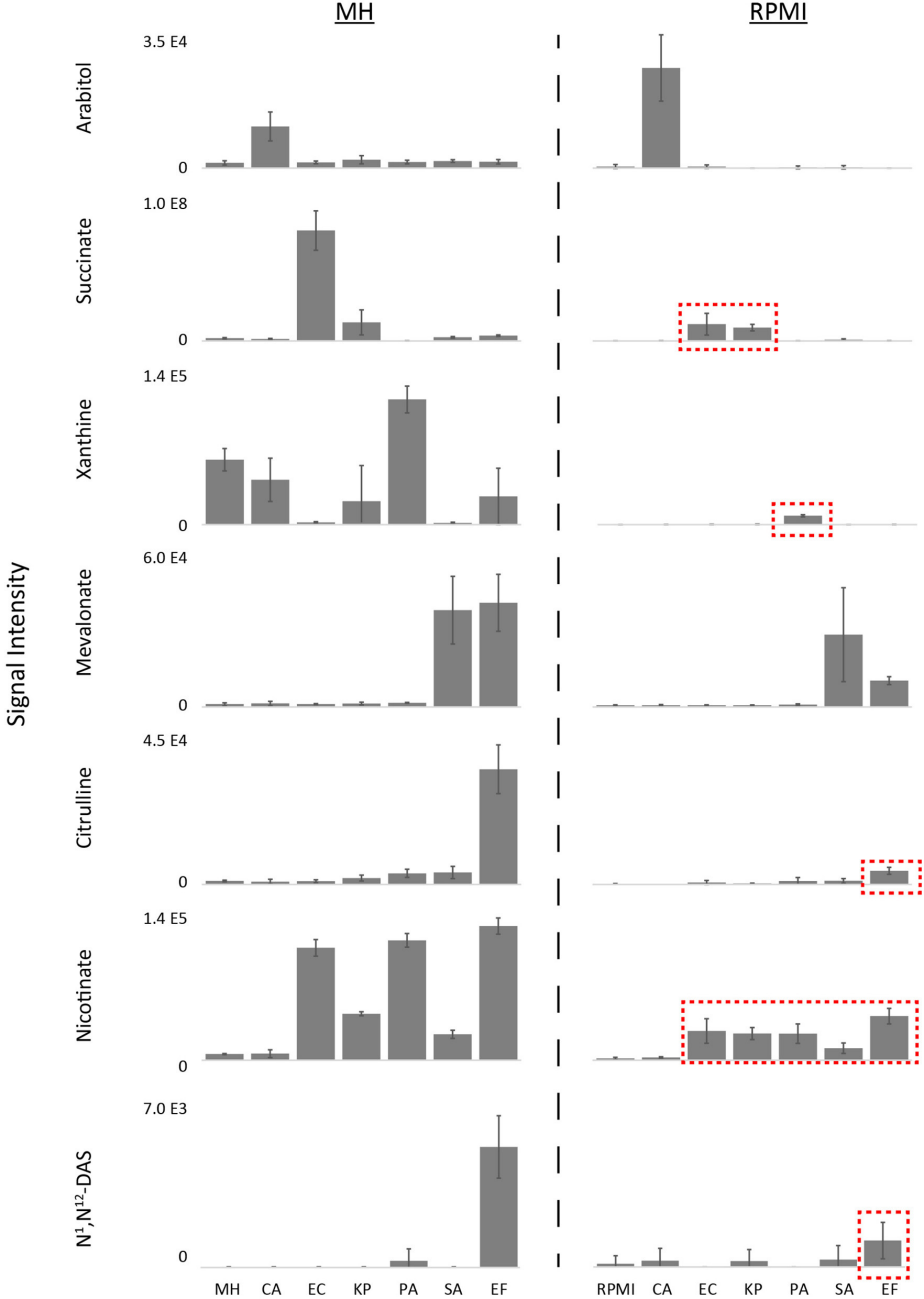
Differential biomarker production of pathogens incubated in MH vs.RPMI medium. While most production biomarkers were observed on MH and RPMI, levels of succinate, xanthine, citrulline, and nicotinate were significantly lower on RPMI, and N^1^,N^12^-diacetylspermine production on RPMI did not meet diagnostic thresholds (Source Data File 3). Key biomarkers that are affected by medium have been highlighted in red boxes. MH, Mueller Hinton medium; RPMI, Roswell Park Memorial Institute medium; CA*, Candida albicans*; KP*, Klebsiella pneumoniae*; EC, *Escherichia coli*; PA*, Pseudomonas aeruginosa*; SA*, Staphylococcus aureus*; EF, *Enterococcus faecalis*. Data are presented as mean values +/− SD of *n* = 4 replicates.

Using a defined medium that is not subject to potential variations in beef or casein sources or downstream production steps is critical for inter-laboratory standardization. We therefore substituted the MH medium with nutrient-rich defined RPMI medium (Supplementary Table 1) to evaluate growth and biomarker production of the six prevalent BSI pathogens tested above. Unsurprisingly, the levels of key nutrients were different between MH medium (BD Lot 1) and RPMI (Supplementary Figure 1, Source Data File 1). Notably, levels of most amino acids, with the exception of glutamine, asparagine, and cysteine, were higher in MH vs. RPMI, whereas glucose and select vitamins were higher in RPMI. While all six microorganisms tested grew on both media, growth was generally poorer on RMPI when compared to MH (Supplementary Figure 2). Furthermore, while most production biomarkers (i.e., arabitol, succinate, xanthine, mevalonate, citrulline, and nicotinate) were still suitable for species differentiation on RPMI, levels of succinate, xanthine, citrulline, and nicotinate were on average 7.5-fold lower when the respective species that produced these markers were grown on RPMI (Figure 3, Source Data File 3). These decreased levels in key biomarkers can have significant impacts on diagnostic accuracy when performing MS/MS fragmentation using less sensitive clinical triple quadrupole mass spectrometers (Groves et al., 2022). Moreover, production of N^1^,N^12^-diacetylspermine—used for identification of EF—was so low on RPMI that it did not meet diagnostic thresholds. The limited production of key biomarkers in RPMI warranted further investigation to identify which medium precursors are responsible for their production in order to potentially improve their levels.

### [U-^13^C]glucose is not a precursor for many biomarkers

**Figure 4:**
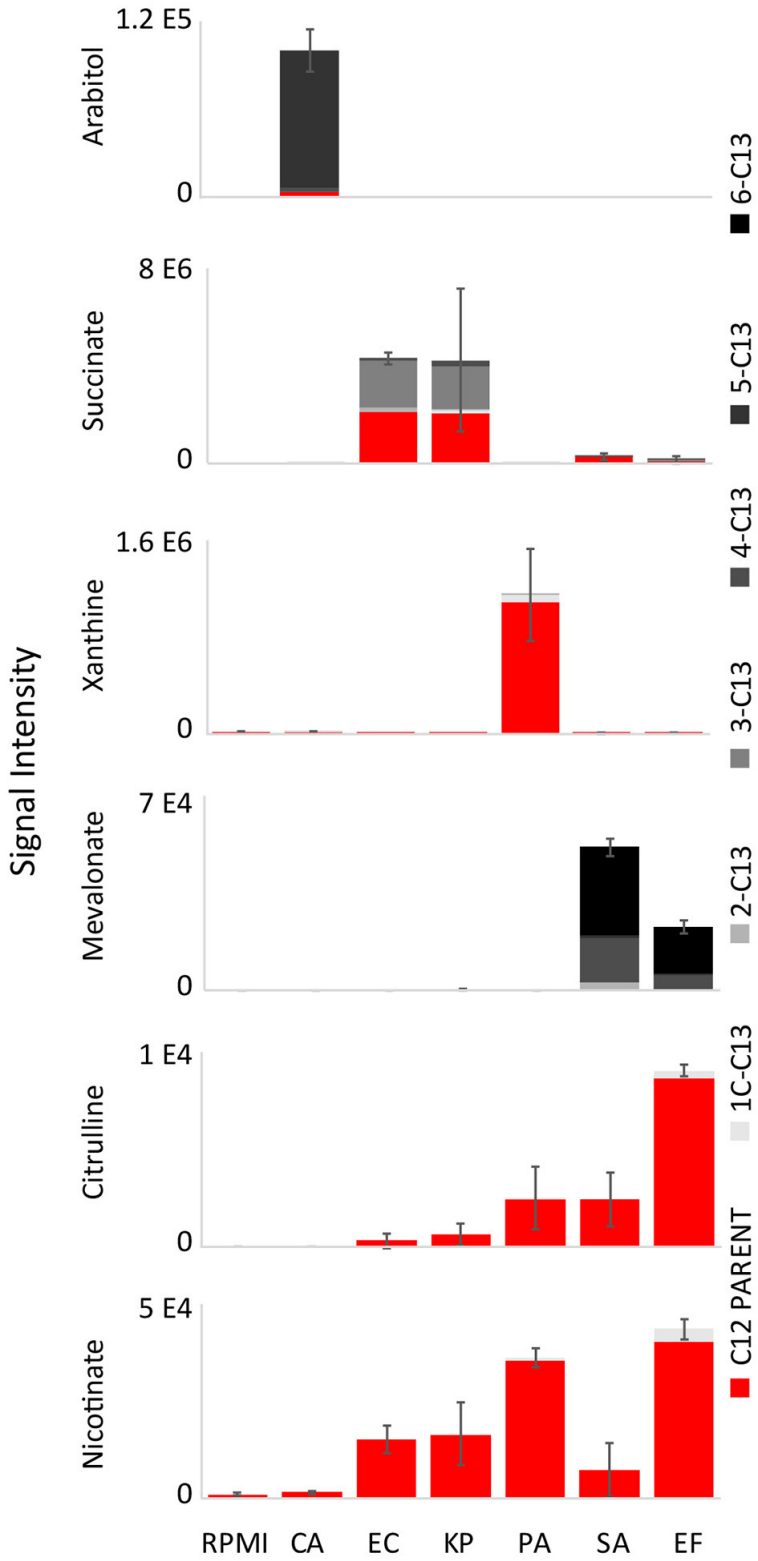
^13^C isotope labeling patterns of top diagnostic biomarkers when isolates were grown on RPMI supplemented with [U-^13^C]glucose. Labeling patterns show that glucose is the primary precursor for arabitol and mevalonate, and partially responsible for succinate production (Source Data File 4). Urocanate, xanthine, citrulline, and nicotinate are fully unlabeled, and thus must be derived from other carbon sources present in the medium. RPMI, Roswell Park Memorial Institute medium; CA*, Candida albicans*; KP*, Klebsiella pneumoniae*; EC*, Escherichia coli*; PA*, Pseudomonas aeruginosa*; SA*, Staphylococcus aureus*; EF, *Enterococcus faecalis*. Data are presented as mean values +/− SD of total signal of *n* = 3 replicates.

To determine if key biomarkers are coming from glucose or other medium components, glucose-free RPMI was supplemented with uniformly labeled [U-^13^C]glucose. The ^13^C isotope labeling patterns of top diagnostic biomarkers (excluding N^1^,N^12^-diacetylspermine) were analyzed using UHPLC-MS (Figure 4, Source Data File 4). Surprisingly, fully unlabeled isotopologues were minimal for arabitol (80% fully ^13^C labeled, and 20% fully unlabeled) and negligible for mevalonate (62% fully ^13^C labeled and 30% ^13^C_4_ labeled), implying that glucose is the primary precursor for these two biomarkers. Succinate was 45% ^13^C_3_ labeled and 49% fully unlabeled. This indicates that a portion of succinate is derived from glucose flowing into the TCA cycle, while the remainder is derived from other precursors. Urocanate, xanthine, citrulline, and nicotinate were fully unlabeled, and thus must be derived from other carbon sources present in the medium.

### Biomarkers were eliminated when precursors were excluded

**Figure 5:**
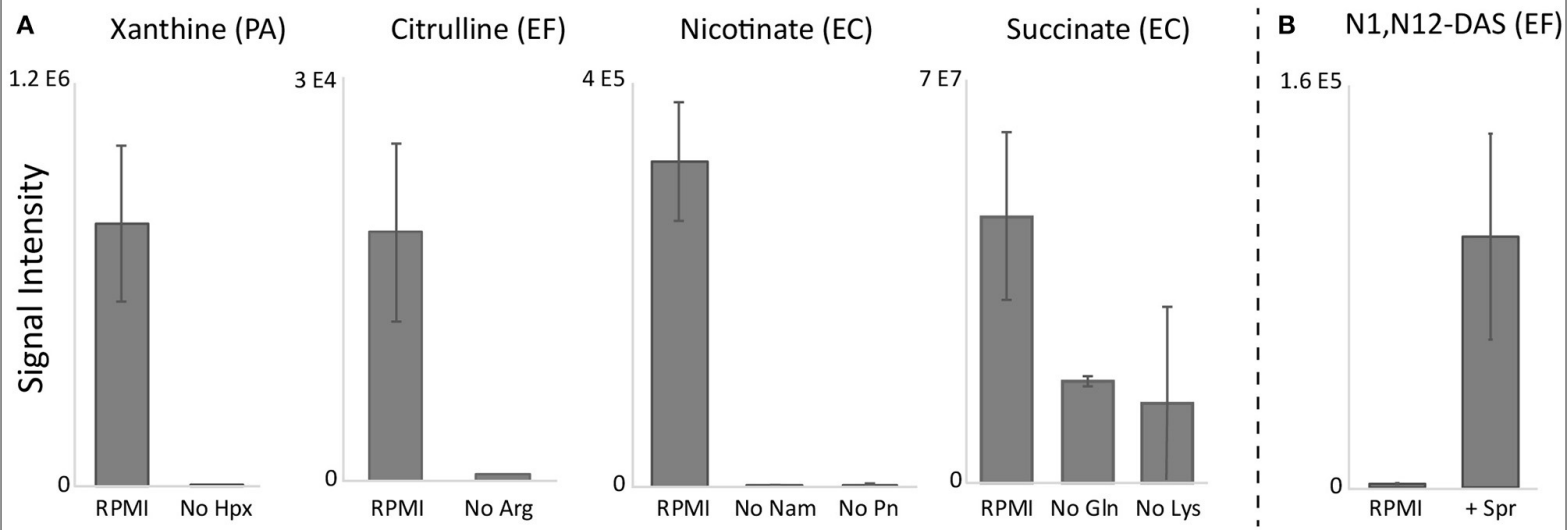
RPMI precursor exclusion **(A)** and supplementation **(B)** results. Exclusion of hypoxanthine (Hpx), arginine (Arg), and nicotinamide (Nam) or pyridoxine (Pn) in RPMI eliminated species-specific production of xanthine, citrulline, and nicotinate, respectively, while exclusion of glutamine (Gln) and lysine (Lys) decreased EC succinate production. Supplementation of RPMI with spermine restored N^1^,N^12^-diacetylspermine (N1,N12-DAS) production. RPMI, Roswell Park Memorial Institute medium; PA*, Pseudomonas aeruginosa*; EF*, Enterococcus faecalis*; EC*, Escherichia coli*. Data are presented as mean values +/− SD of *n* = 3 replicates.

To identify likely precursors for biomarkers that did not predominantly come from glucose (i.e., citrulline, xanthine, nicotinate, and succinate) we excluded the individual amino acids and other putative precursors found in RPMI and assessed the effect on these four biomarkers. We took a two-pronged approach for these experiments: we both randomly screened individual exclusion of the majority of amino acids found in RPMI (Source Data File 5), and investigated pathways that could provide insights into which substrates could be contributing to biomarker production (Wishart et al., 2018; Karp et al., 2019). While it was difficult to identify which precursors could be partially responsible for the additional succinate production, precursors for other biomarkers could be deduced from metabolic pathways. For example, xanthine could potentially be derived from hypoxanthine—a component of RPMI—*via* xanthine oxidase (EC:1.17.3.2) which is present in the adenine dinucleotide salvage pathway (Bortolotti et al., 2021). Citrulline has been proposed to be formed from arginine *via* nitric-oxide synthase (EC:1.14.13.39) or indirectly *via* the urea cycle (Marletta, 1993). Nicotinate could be derived from nicotinamide, also present in RPMI, *via* nicotinamidase (EC:3.5.1.19) present in the nicotinamide adenine dinucleotide salvage pathway (Berger et al., 2004). Indeed, exclusion of hypoxanthine in PA cultures, arginine in EF cultures, and nicotinamide in EC (and other) cultures eliminated production of citrulline, xanthine, and nicotinate, respectively (Figure 5A, Source Data File 5). Notably, exclusion of pyridoxine, a known co-enzyme used in aminotransferase reactions (Feldman and Gunsalus, 1950; Gunsalus and Tonzetich, 1952) in EC cultures also eliminated nicotinate production. Lastly, screening of amino acid exclusions revealed that exclusion of both glutamine and lysine decreased succinate production in EC cultures. Given that N^1^,N^12^-diacetylspermine production was negligible in RPMI for EF cultures, we suspected that the precursor for this biomarker may be absent in RPMI. Through pathway analysis, we hypothesized that spermine supplementation could restore N^1^,N^12^-diacetylspermine secretion in EF cultures (Pegg, 2008). Indeed, supplementation of RPMI with spermine not only restored N^1^,N^12^-diacetylspermine production by EF, but also resulted in N^1^,N^12^-diacetylspermine production in EC and KP cultures (Figure 5B, Source Data File 5).

### Non-glucose biomarker precursors were confirmed using labeled precursors

**Figure 6:**
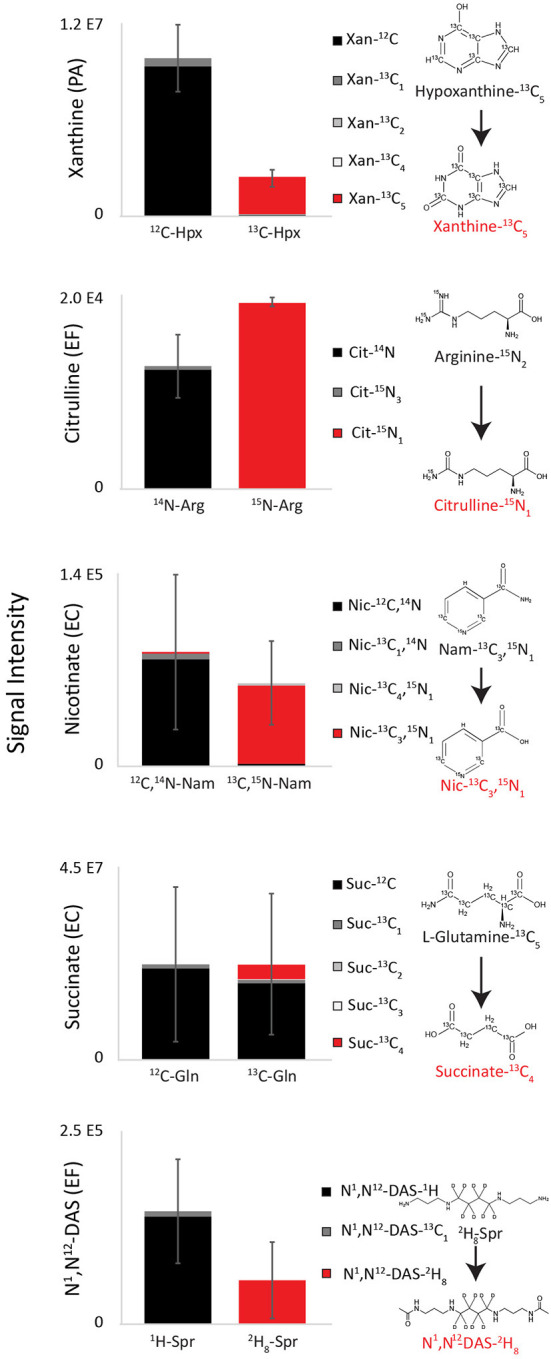
Labeling patterns of top diagnostic biomarkers when isolates were grown on RPMI supplemented with putative precursors. Species-specific xanthine, citrulline, nicotinate, and N^1^,N^12^-diacetylspermine were all >95% labeled when labeled substrates were used, demonstrating that that hypoxanthine, arginine, nicotinamide, and spermine are direct medium precursors for xanthine, citrulline, nicotinate, and N^1^,N^12^-diacetylspermine (Source Data File 6). EC cultured in RPMI with unlabeled glucose and uniformly labeled L-glutamine-^13^C_5_ was 80% fully ^12^C labeled and 15% fully ^13^C_4_ labeled, demonstrating that it is mostly synthesized from glucose, although glutamine partially contributes to its production as well. Hpx, Hypoxanthine; Arg, arginine; Nam, nicotinamide; Gln, glutamine; Spr, spermine; PA*, Pseudomonas aeruginosa*; EF*, Enterococcus faecalis*; EC*, Escherichia coli*. Data are presented as mean values +/− SD of *n* = 3 replicates.

To further confirm species-specific biomarker precursors identified in precursor exclusion experiments, unlabeled precursors were substituted with labeled analogs in RPMI medium (Figure 6, Source Data File 6). Resulting species-specific xanthine, citrulline, nicotinate, and N^1^,N^12^-diacetylspermine were all >95% labeled when labeled substrates were used. Specifically, reduction of uniformly labeled hypoxanthine-^13^C_5_ in PA cultures resulted in uniformly labeled xanthine-^13^C_5_. Deamination of arginine (Guanido-^15^N_2_) in EF cultures resulted in citrulline-^15^N_1_. Substitution of the terminal amine with a hydroxide of nicotinamide-2,6,7-^13^C_3_-(pyridyl-^15^N) resulted in nicotinate-2,6,7-^13^C_3_-(pyridyl-^15^N). This data suggests that hypoxanthine, arginine, and nicotinamide are direct medium precursors for xanthine, citrulline, and nicotinate, respectively. Similarly, deacetylation of spermine-(butyl-d8) tetrahydrochloride resulted in N^1^,N^12^-diacetylspermine (butyl-d8). Succinate produced by EC cultured in RPMI with unlabeled glucose and uniformly labeled L-glutamine-^13^C_5_ was 80% fully ^12^C labeled and 15% fully ^13^C_4_ labeled. This result confirms that succinate is mostly synthesized from glucose, although glutamine partially contributes to its production as well.

### Chemically defined biomarker enrichment medium restored pathogen metabolic phenotypes to those that were originally observed in Mueller Hinton

Our ultimate objective was to develop a reproducible defined medium allowing for pathogen growth and that enabled the production of the key biomarkers originally observable in MH medium (Rydzak et al., 2022). Using defined RPMI as our basal medium, and integrating the additional precursors needed for select biomarkers, we developed a modified, defined Biomarker Enrichment Medium (BEM; Supplementary Table 1). A number of critical modifications were made to RPMI to generate BEM (see Supplementary Table 1 for all modifications). (1) Medium was supplemented with additional amino acids that were found in MH and not present in RPMI. (2) Concentrations of many amino acids were increased to more closely resemble those found in MH medium. (3) Trace metals and nucleosides were also added to potentially promote growth of a wider array of species. (4) Catalase was added to promote the viability of species that produce hydrogen peroxide (ex. *Streptococcus pneumoniae*). (5) Albumax (a lipid-rich bovine serum albumin formulation), which is commonly added to RPMI as a substitute for human serum, was eliminated to ensure that BEM is fully defined. (6) While sucrose was added in addition to glucose, concentrations of total sugars was kept low (i.e., 5-fold hexose equivalents lower than original RPMI) to promote metabolism of amino acids and other components during the short, 4 h incubation period. (7) BEM was supplemented with spermine and concentrations of other identified precursors (with the exception of nicotinamide) were increased.

**Figure 7:**
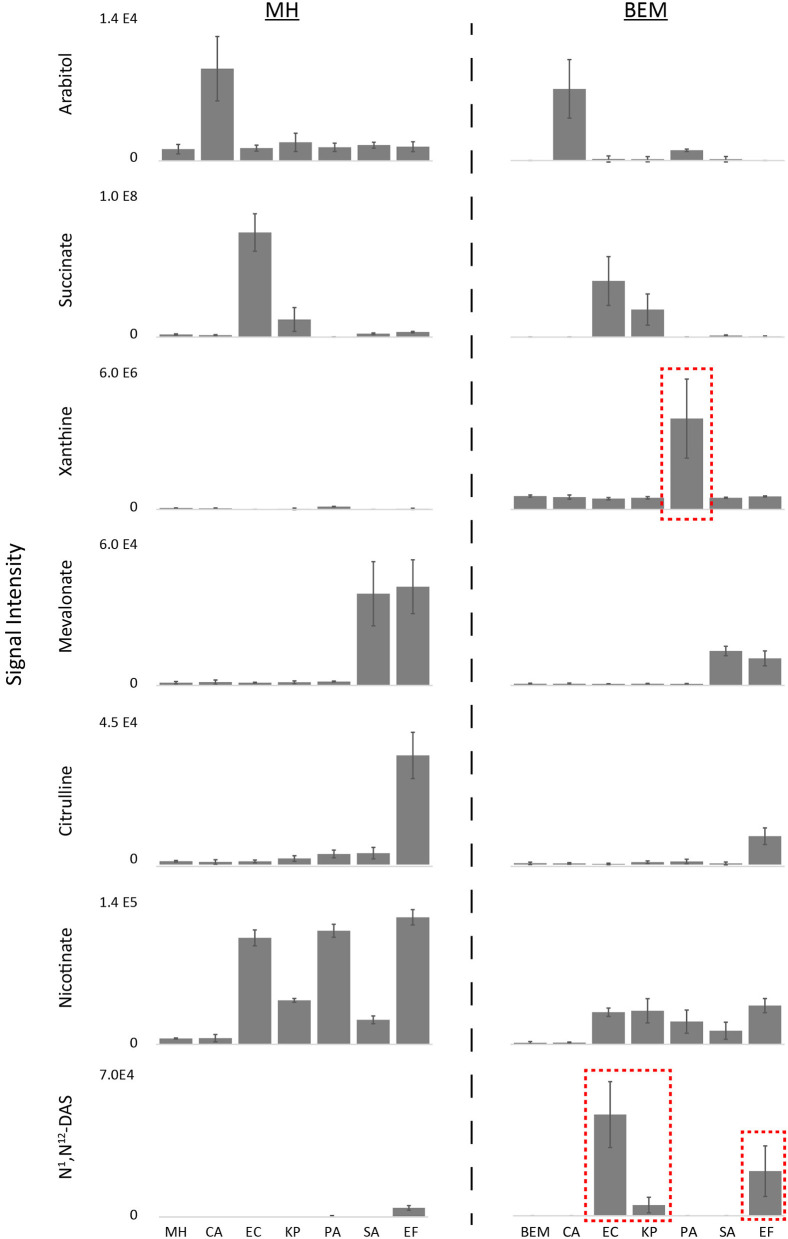
Differential biomarker production of pathogens incubated in MH vs. defined BEM medium. Metabolic phenotypes of MH were reproduced in BEM. Notably, supplementation of hypoxanthine in BEM improved PA xanthine levels and addition of spermine resulted in improved N^1^,N^12^-diacetylspermine production by EF, as well as EC and KP (Source Data File 3). Key biomarkers that are affected by medium have been highlighted in red boxes. MH, Mueller Hinton medium; BEM, Biomarker Enrichment Medium; CA*, Candida albicans*; KP*, Klebsiella pneumoniae*; EC*, Escherichia coli*; PA*, Pseudomonas aeruginosa*; SA*, Staphylococcus aureus*; EF, *Enterococcus faecalis*. Data are presented as mean values +/− SD of *n* = 3 replicates.

Our final BEM medium sustained growth of all six pathogen tested (*n* = 3). While growth of EC, KP, and SA was best on MH, potentially due to a richer nutrient composition, growth of these species was comparable on RPMI and BEM (Supplementary Figure 2). CA and EF grew similarly on all three media (except one EF isolate on MH), while PA grew well on MH and BEM, but not on RPMI. More importantly, our defined BEM produced the necessary diagnostic biomarkers originally identified in MH medium (Figure 7, Source Data File 3). While strictly glucose-derived biomarkers (arabitol and mevalonate) were lower on BEM than MH and RPMI (1.3 and 3.3-fold lower for CA arabitol production and 2.6 and 2.0-fold lower for SA/EF mevalonate production, respectively), they were still significantly higher than the signals from other cultures (≥7.0-fold higher for arabitol and ≥16.4-fold higher for mevalonate), and thus had sufficient diagnostic power. EC and KP succinate production was comparable to MH and 2.2 to 3.5-fold higher than that observed in RPMI, potentially due to higher glutamine and lysine concentrations. Nicotinate production by EC, KP, PA, SA, and EF was almost identical to that of RPMI, which was unsurprising, given that nicotinamide concentrations were the same in both media. While EF citrulline concentrations were 3.9-fold lower in BEM when compared to MH, increasing arginine concentrations increased citrulline concentrations by 2.2-fold when compared to RPMI. Most importantly, supplementation of hypoxanthine in BEM increased PA xanthine levels by 33 and 467-fold when compared to MH and RPMI, respectively. Similarly, addition of spermine restored N^1^,N^12^-diacetylspermine production by EF, production that was almost absent in RPMI. Notably, addition of spermine also resulted in N^1^,N^12^-diacetylspermine production by EC and KP. However, EF can still be differentiated from EC and KP by the absence of succinate production.

### Biomarker patterns for antibiotic susceptibility testing using BEM and clinically approved MH medium are comparable

**Figure 8:**
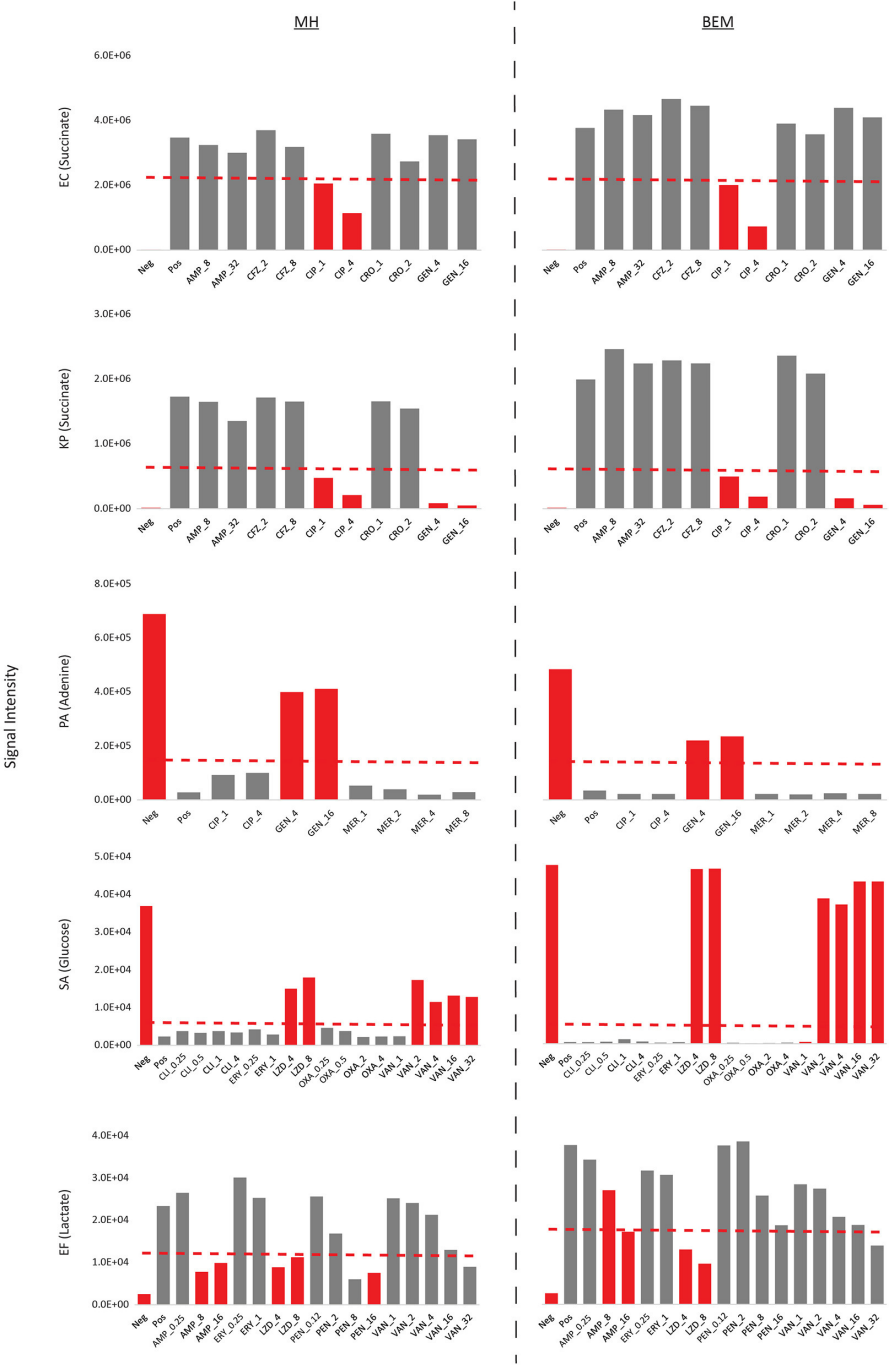
Antibiotic susceptibility validation on BEM and MH II using metabolic inhibition profiles. Metabolic inhibition of select biomarkers in response to antibiotics were tested on bacterial isolates with known microdilution or Vitek2 AST profiles. Inhibition of biomarker production/consumption were in general agreement with microbroth dilution/Vitek2 calls, correctly identifying sensitive (gray bars) and resistant strains (red bars). Furthermore, changes in biomarker levels in both media were comparable. Sensitive vs. resistant threshold cutoffs are indicated in dashed red lines. Concentrations in antibiotics are provided as μg/mL. Neg, uninoculated medium control; Pos, inoculated control with no added antibiotics; AMP, ampicillin; CFZ, cefazolin; CIP, ciprofloxacin; CRO, ceftriaxone; GEN, gentamicin; MER, meropenem; CLI, clindamycin; ERY, erythromycin; LZD, linezolid; OXA, oxacillin; VAN, vancomycin; PEN, benzylpenicillin.

To ensure that BEM is suitable for antibiotic susceptibility testing (AST), we measured the metabolic inhibition of select biomarkers in response to commonly prescribed antibiotics on bacterial isolates with known microdilution or Vitek2 AST profiles using a rapid MS/MS assay (see Methods; Source Data File 8, Figure 8). Inhibition of succinate production, adenine consumption, glucose consumption, and lactate production were used to assess antibiotic efficacy on EC and KP, PA, SA, and EF, respectively, on both BEM and CLSI approved MH II. For EC, KP, PA, and SA, inhibition of biomarker production/consumption not only identified sensitive (gray bars) and resistant strains (red bars) accurately in agreement with microbroth dilution methods (or Vitek2 for SA) in both media, but changes in biomarker levels in both media were comparable. Furthermore, sensitive vs. resistant threshold cutoffs (dashed red lines) for these species could be set at identical levels when species are incubated on either BEM or MH II. Some discrepancies between metabolic inhibition assay (MIA) results and Vitek2 results were observed for EF. Specifically, the MIA on MH II identified EF as sensitive to 8 μg/mL of benzylpenicillin and 32 μg/mL of vancomycin, contradicting Vitek2 results. Similarly, the MIA on TM identified EF as resistant to 8 μg/mL of ampicillin and sensitive to 32 μg/mL of vancomycin in contradiction to Vitek2 results. Minor adjustments in cation levels may address these discrepancies. However, one serial dilution differences between platforms are generally acceptable by regulatory bodies (Food Administration, 2007). Ideally, MIA results should be compared to microbroth dilution results. While identical sensitive vs. resistant threshold cutoffs could be used for most species on both media, these thresholds needed to be re-calibrated for each medium with respect to lactate production levels for EF. This may be attributed to small changes in growth and/or metabolism on each medium for EF. Nevertheless, we show here that BEM is not only effective at identifying species based on metabolic patterns, but also that it can be effectively used to differentiate sensitive vs. resistant strains based on metabolic inhibition profiles of select metabolites and that these profiles are comparable to those of MH II medium.

## Discussion

In this study, we showed that batches of clinical MH have significant lot-to-lot variability in their small molecule composition. This variability, in turn, had a direct impact on the metabolic phenotypes observed across a broad range of opportunistic pathogens when cultured *in vitro*. Moreover, these changes had a significant impact on our ability to differentiate these pathogens using metabolomics approaches. To address this, we developed a defined biomarker enrichment medium (BEM) that was specifically tuned for enhancing the diagnostic metabolic phenotypes of bloodstream pathogens and to enable robust to lot-to-lot reproducibility of these phenotypes.

Although we developed BEM with clinical applications in mind, our observations about the instability of metabolic phenotypes elicited by undefined media and our strategy for resolving these problems are generalizable to a broad range of other metabolomics-based applications. Metabolomics is emerging as a mainstream microbiology tool that is seeing increasing use in clinical diagnostics (Kennedy et al., 2018), metabolic engineering (Dromms and Styczynski, 2012), and is becoming a key tool for unraveling complex host-microbe dynamics (Han et al., 2010). The variability in metabolic phenotypes that we observed here could negatively affect any of these applications and may have a direct impact on our ability to reproduce metabolomic studies lab-to-lab. The strategy we introduce here (substrate exclusion, isotope tracing, and mass spectrometry-based metabolic analyses) for identifying substrate-product relationships, and enriching desirable phenotypes, is broadly applicable to any *in vitro* culture system and may help researchers develop other specialized media to ensure their experiments can be replicated by other groups.

In addition to stabilizing metabolic phenotypes, developing defined growth media has a range of other benefits. For instance, many efforts have gone into developing defined media in order to sustain growth of microorganisms (Cocaign-Bousquet et al., 1995; Letort and Juillard, 2001; Zhang et al., 2009), improve downstream purification steps of industrially valuable secreted proteins [ex. bacteriocins; (Mah et al., 1967; Pingitore et al., 2009; Khan et al., 2013)], and enable the analysis of secreted compounds [ex. exopolysaccharides; (Grobben et al., 1998; Torino et al., 2005)]. The nutrient composition of a defined medium also influences production of secreted compounds. For example, vitamins and osmolites have been shown to impact growth and bacteriocin production in *Lactobacillus salivarius*, adenine and lactose can stimulate exopolysaccharide production in *Lactobacillus helveticus*, higher CaCl_2_ and low magnesium concentrations improved bioethanol yields in *Clostridium thermocellum*, and pentose sugars can inhibit growth and biofuel yields (Torino et al., 2005; Pingitore et al., 2009); Vera (Pingitore et al., 2009; Girardello et al., 2012b; Islam et al., 2013; Sander et al., 2015; Verbeke et al., 2017). Medium cation concentrations of different MH agar brands can also influence antibiotic susceptibility results (Girardello et al., 2012a). Furthermore, the cleaner matrix of chemically defined medium can also improve the resolution of mass spectrometry analysis (Texeira et al., 2015). In summary, chemically defined media have a range of benefits beyond metabolism that warrant consideration in any large-scale studies.

One important caveat to the findings reported here is that the significant media-related variability we observed in metabolic phenotypes may not necessarily translate to more traditional clinical microbiology assays. MH is a Clinical and Laboratory Standards Institute (CLSI) approved medium for growth of pathogens and has a multi-decade track record as the gold standard media for clinical microbiology (Clinical and Laboratory Standards Institute, 2022). The distinction between our findings and this previously established efficacy is attributable to the difference between the two approaches: metabolomics monitors the concentrations of individual nutrients, whereas clinical assays generally track microbial growth. Since microbes can satisfy their biomass and energetic needs using a variety of precursors (Koch, 2005), minor variations in the levels of individual nutrients will not necessarily be reflected in altered *in vitro* growth rates.

In conclusion, we have shown that undefined media can be an unstable foundation for biomarker discovery, clinical diagnostics, and other metabolism-based microbiology assays where reproducibility is paramount. We show that our specifically engineered BEM reduces batch-to-batch variation and optimizes our target metabolic phenotypes. We recommend that future metabolomics-based studies carefully consider chemical composition of the growth media.

## Materials and methods

### Bacterial strains, cultivation, and sample preparation

*Candida albicans, Klebsiella pneumoniae, Escherichia coli, Pseudomonas aeruginosa, Staphylococcus aureus*, and *Enterococcus faecalis* used in for biomarker identification experiments were recovered from patient blood culture samples and provided as cryo stocks by Alberta Precision Laboratories (APL) and approved by the Conjoint Regional Ethics Board (REB # 16-2457 and REB#17-1525, UC). Isolates used for antibiotic susceptibility testing (AST) with known antibiotic susceptibility profiles were obtained from the Center for Disease Control (EC SAMN04014991, KP SAMN04014967, PA SAMN04014936) or from the American Type Culture Collection (SA ATCC BAA-1708, EF ATCC 51575). Antibiotic susceptibility profiles of ATCC strains were determined using the Vitek2 (BioMérieux) according to manufacturer protocols. All strains were cultured in a humidified incubator (Heracell VIOS 250i Tri-Gas Incubator, Thermo Scientific, Waltham, Mass. USA) under a 5% CO_2_ and 21% O_2_ atmosphere. Cryo stocks were first revived on Mueller Hinton agar plates overnight. Colonies were then scraped off the plate and diluted to a 0.5 McFarland (OD_600_ ~ 0.15 or ~7.5 x 10^7^ CFU/mL) in respective medium (see below), and incubated for 4 h as described previously (Rydzak et al., 2022). Cultures were then centrifuged for 10 min for 4,000 g at 4°C to remove cells. Supernatant was removed, mixed 1:1 with 100% LC-MS grade methanol, and frozen at−80°C for further processing. Upon thawing, samples were centrifuged again for 10 min at 4,000 g at 4°C to remove any protein precipitate and supernatants were diluted 1:10 with 50% LC-MS grade methanol and analyzed using UHPLC-MS.

### Chemicals and medium preparation

All chemicals were obtained from Sigma-Aldrich (St. Louis, Mo. USA), VWR (Radnor, Pa. USA), or Fisher Scientific (Waltham, Mass. USA) unless otherwise specified. To study the lot-to-lot variability in MH, different lots of media was sourced from BD (Becton, Dickinson, and Co) and Fluka Analytical. These include MH I from BD (BD lots 1-3; 1025007, 8119710, 9127956), MH I from Fluka Analytical (Fluka Lot 1-2; BCBG7588V, BCBM5081V), and MH II from BD (BD Lot 4-6; 8095574, 8190586, 9044411). Each medium was prepared according to the recipe specified on the bottle, autoclaved for 20 min at 121°C, and stored at 4°C. RPMI 1640 was supplemented with 25 mM HEPES, 100 μM hypoxanthine, and 2.5 g/L Albumax II [see Supplementary Table 1 for full recipe; (Lewis et al., 2014)] and filter sterilized (pore size, 0.2 μm; Millipore, Billerica, MA). Final Biomarker Enrichment Medium composition is also provided in Supplementary Table 1. Catalase was added to medium on the same day as experiments were performed, and medium was filter sterilized.

To investigate which biomarkers were derived from glucose, RPMI 1640 medium with no glucose (MP Biomedicals, 1646854) was supplemented with uniformly labeled [U-^13^C]glucose purchased from Cambridge Isotope Laboratories (catalog number: CLM-1396-50). To identify likely precursors for biomarkers that did not predominantly come from glucose (i.e., citrulline, xanthine, nicotinate, and succinate) we screened the effect of exclusion of individual amino acids and other putative precursors found in RPMI on the production of these four biomarkers. RPMI was made according to the recipe in Supplementary Table 1 and amino acids and other putative precursors/cofactors (nicotinamide, hypoxanthine, pyridoxine) were excluded individually.

To verify species-specific biomarker precursors, L-arginine, nicotinamide, spermine, L-glutamine, and hypoxanthine in RPMI were individually replaced with labeled analogs. Arginine-HCl (Guanido-^15^N_2_) (catalog number: NLM-395-1), hypoxanthine (^13^C_5_) (catalog number: CLM-8042-0.1MG), and L-glutamine (^13^C_5_) (catalog number: CNLM-1275-H-0.5) were purchased from Cambridge Isotope Laboratories. Spermine-(butyl-d8) tetrahydrochloride (catalog number: 705330-5MG) was purchased from Sigma-Aldrich. Finally, nicotinamide-2,6,7-^13^C_3_-(pyridyl-^15^N) (catalog number: 809799-1MG) was purchased from Millipore Sigma.

### Growth measurements

Growth curves were performed in 96-well-plates incubated inside a spectrophotometer (Multiskan Go, Thermo Scientific) at 37°C for 24 h. Turbidity was measured every hour at 600 nm. Optical density values were adjusted to a 1 cm pathlength by multiplying the net change in OD_600_ by a conversion factor of 4.6. Given that growth curves were performed for 24 h, evaporation occurring in the wells near the edge of the 96-well-plate could affect optical density. To avoid this technical error, all the wells near the edges were filled will non-inoculated medium, and only central wells were inoculated.

### UHPLC-MS

All metabolomics data were acquired at the Calgary Metabolomics Research Facility (CMRF) as previously described (Rydzak et al., 2022). Briefly, chromatography was performed using hydrophilic interaction liquid chromatography (HILIC) with changing gradients of 20 mM ammonium formate at pH 3.0 in LC-MS grade water (Solvent A) and 0.1% formic acid (% v/v) in LC-MS grade acetonitrile (Solvent B) on a Thermo Fisher Scientific Vanquish UHPLC platform. The flow rate used in all analyses was 600 μL/min and the sample injection volume was 2 μL. Samples were analyzed in both negative (−2,000 V) and positive mode (+3,000 V) on a Thermo Scientific Q ExactiveTM HF (Thermo Scientific) mass spectrometer using full scan acquisitions (50–750 *m/z*) with a 240,000 resolving power, an automatic gain control target of 3e6, and a maximum injection time of 200 ms. Data for antibiotic susceptibility testing validation were acquired on a TSQ Altis Triple Quadrupole (Thermo Scientific) mass spectrometer operating in selected reaction monitoring (SRM) mode using flow injection analysis. Samples were run at a flow rate of 1.5 mL/min using 60/40 LC-MS grade acetonitrile/water (%v/v) with 0.1% formic acid (%v/v) running isocratically with a method duration of 0.65 mins. Samples were run in positive (+3,000V) and negative (−2,500V) ion mode using the following SRM transitions with their respective collision energies (CE): adenine [M+H]^+^ 136 *m/z*-> 119 *m/z* (23V CE), lactate [M-H]^−^ 89 *m/z*-> 43 *m/z* (12V CE), succinate [M-H]^−^ 117 *m/z*-> 73 *m/z* (11V CE), and glucose [M+Cl]^−^ 215 *m/z*-> 35 *m/z* (11V CE).

### Computational methods

All MS analyses were conducted in MAVEN (El-MAVEN v0.12.0) (Melamud et al., 2010). Untargeted analysis of different batches of MH medium was performed with the peak picking function in El-MAVEN v0.12.0 with a 10 ppm *m/z* window and a minimum peak intensity set to 50,000. Targeted peak assignments were based on the Mass Spectrometry Metabolite Library of Standards (MSMLS, IROA Technologies) as previously described (Groves et al., 2022). Statistical analyses were conducted using the R statistical software platform (R Core Team, 2022) using in-house software tools previously published [https://zenodo.org/record/6403220#.YkYXoi9730o; (Rydzak et al., 2022)].

## Data availability statement

The original contributions presented in the study are included in the article/Supplementary Material, further inquiries can be directed to the corresponding author/s.

## Author contributions

MM, TR, RG, and IL designed the experiments. MM and TR performed experiments. RG, MM, and TR collected and interpreted mass spectrometry data. TR, MM, and IL wrote the manuscript. All authors contributed to the article and approved the submitted version.

## Funding

This work was supported by Genome Canada's 2016 Genomics Application Partnership Program (GAPP) award and the 2017 Large Scale Applied Research Project (LSARP) competition, administered through Genome Alberta. This work was made possible in part by a research collaboration agreement with Thermo Fisher. IL was supported by an Alberta Innovates Translational Health Chair. TR was supported by an Eyes High Postdoctoral Fellowship from the University of Calgary. Metabolomics data were acquired at the Calgary Metabolomics Research Facility, which was supported by the International Microbiome Center and the Canada Foundation for Innovation (CFI-JELF 34986). This research was funded by a Genome Canada GAPP award, which is intended to enable the commercialization of research findings.

## Conflict of interest

IL and TR have submitted a patent (International Application Number CA2019051351) describing substrate-product relationships used to identify pathogens. The remaining authors declare that the research was conducted in the absence of any commercial or financial relationships that could be construed as a potential conflict of interest.

## Publisher's note

All claims expressed in this article are solely those of the authors and do not necessarily represent those of their affiliated organizations, or those of the publisher, the editors and the reviewers. Any product that may be evaluated in this article, or claim that may be made by its manufacturer, is not guaranteed or endorsed by the publisher.

## Supplementary material

Supplementary Figure 1 Comparison of MH and RPMI medium components. Levels of most amino acids, with the exception of glutamine, asparagine, and cysteine, were higher in MH vs. RPMI, whereas glucose and select vitamins were higher in RPMI.

Supplementary Figure 2 Growth rates on MH, RPMI, and BEM medium. Biological replicates (*n* = 3; red, blue and gray lines) of *Candida albicans* (CA), *Klebsiella pneumoniae* (KP), *Escherichia coli* (EC), *Pseudomonas aeruginosa* (PA), *Staphylococcus aureus* (SA), *Enterococcus faecalis* (EF), and *Streptococcus pneumoniae* (SP) were grown for 24 h.

Supplementary Table 1 Composition of RPMI and BEM.
