# Supplementary figures and images for: Biomarker enrichment medium: A defined medium for metabolomic analysis of microbial pathogens
Source: Front Microbiol. 2022 Jul 22;13:957158. doi: 10.3389/fmicb.2022.957158 (PMC9354526; doi:10.3389/fmicb.2022.957158)

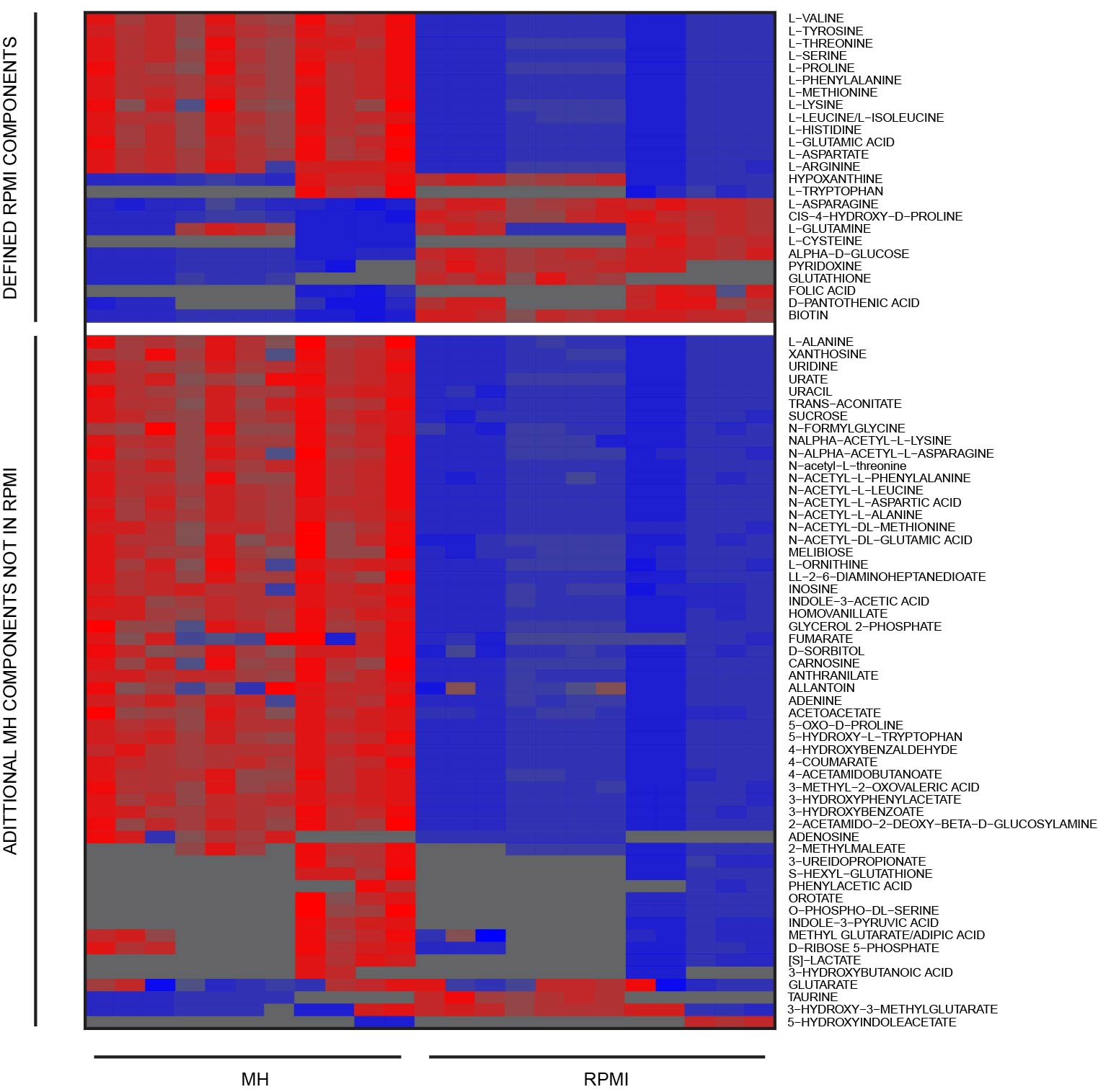

Supplement: Supplementary Figure 1 — Comparison of MH and RPMI medium components. Levels of most amino acids, with the exception of glutamine, asparagine, and cysteine, were higher in MH vs. RPMI, whereas glucose and select vitamins were higher in RPMI. [file Image_1.JPEG]

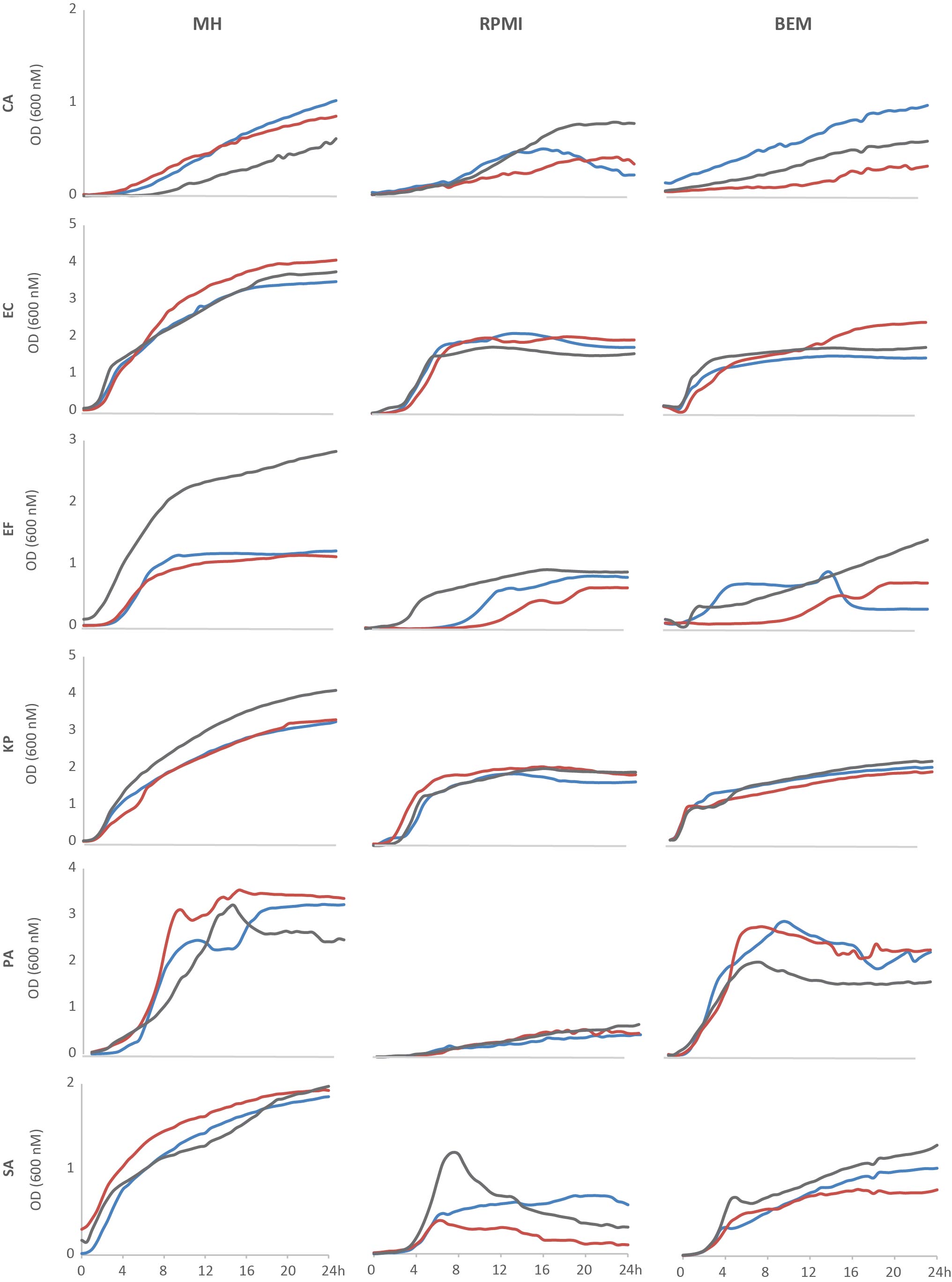

Supplement: Supplementary Figure 2 — Growth rates on MH, RPMI, and BEM medium. Biological replicates (n = 3; red, blue and gray lines) of Candida albicans (CA), Klebsiella pneumoniae (KP), Escherichia coli (EC), Pseudomonas aeruginosa (PA), Staphylococcus aureus (SA), Enterococcus faecalis (EF), and Streptococcus pneumoniae (SP) were grown for 24 h. [file Image_2.JPEG]
