# Supplementary material for: Biomarker enrichment medium: A defined medium for metabolomic analysis of microbial pathogens
Source: Front Microbiol. 2022 Jul 22;13:957158. doi: 10.3389/fmicb.2022.957158 (PMC9354526; doi:10.3389/fmicb.2022.957158)
Supplement: Supplementary Table 1 — Composition of RPMI and BEM. [file Table_1.DOCX]

| **Supplementary Table 1.** Composition of RPMI and BEM. Compounds required for select markers are italicized. | | | | | | | | | |
| --- | --- | --- | --- | --- | --- | --- | --- | --- | --- |
| **Compound** | **BEM** | | **RPMI** | | **Compound** | **BEM** | | **RPMI** | |
|  | **g/L** | **mM** | **g/L** | **mM** |  | **g/L** | **mM** | **g/L** | **mM** |
| Alanine | **1** | 11.22 | N/A | N/A | FeSO4 • 6H2O | **2.60E-04** | 1.00E-03 | N/A | N/A |
| Cysteine • HCl | **0.5** | 3.17 | N/A | N/A | CuSO4 • 5H2O | **2.50E-06** | 1.00E-05 | N/A | N/A |
| Glycine | 0.01 | 0.13 | 0.01 | 0.13 | ZnSO4 • 7H2O | **2.88E-06** | 1.00E-05 | N/A | N/A |
| ***Hypoxanthine*** | **0.25** | 1.84 | 1.36E-02 | 0.10 | MnCl2 • 4H2O | **1.58E-05** | 8.00E-05 | N/A | N/A |
| Hydroxy-L-Proline | N/A | N/A | 0.02 | 0.15 | H3BO3 | **2.47E-05** | 4.00E-04 | N/A | N/A |
| ***L-Arginine*** | **1** | 5.74 | 0.2 | 1.15 | CoCl2 • 6H2O | **7.14E-06** | 3.00E-05 | N/A | N/A |
| L-Asparagine (anhydrous) | 0.05 | 0.38 | 0.05 | 0.38 | Choline Chloride | 0.003 | 2.10E-02 | 0.003 | 2.15E-02 |
| L-Aspartic Acid | **1** | 7.51 | 0.02 | 0.15 | D-Biotin | 2.00E-04 | 8.20E-04 | 2.00E-04 | 8.19E-04 |
| L-Cystine • 2HCl | 0.05 | 0.16 | N/A | N/A | D-Pantothenic Acid (hemicalcium) | 2.50E-04 | 1.00E-03 | 2.50E-04 | 1.00E-03 |
| L-Glutamic Acid | **3** | 20.39 | 0.02 | 0.14 | Folic Acid | 0.001 | 2.30E-03 | 0.001 | 2.27E-03 |
| ***L-Glutamine*** | **5** | 34.21 | 0.3 | 2.05 | Glutathione (reduced) | 0.001 | 3.30E-03 | 0.001 | 3.25E-03 |
| L-Histidine | **0.5** | 3.22 | 0.015 | 0.10 | myo-Inositol | **N/A** | N/A | 0.035 | 1.94E-01 |
| L-Isoleucine | **1** | 7.62 | 0.05 | 0.38 | ***Nicotinamide*** | 0.001 | 8.20E-03 | 0.001 | 8.19E-03 |
| L-Leucine | **1** | 7.62 | 0.05 | 0.38 | p-Aminobenzoic Acid | 0.001 | 7.30E-03 | 0.001 | 7.29E-03 |
| ***L-Lysine • HCl*** | **5** | 27.38 | 0.04 | 0.22 | Pyridoxine • HCl | 0.001 | 4.90E-03 | 0.001 | 4.90E-03 |
| L-Methionine | **0.5** | 3.35 | 0.015 | 0.10 | Riboflavin | 2.00E-04 | 5.30E-04 | 2.00E-04 | 5.31E-04 |
| L-Ornithine | **0.01** | 0.08 | N/A | N/A | Thiamine • HCl | 0.001 | 3.00E-03 | 0.001 | 3.00E-03 |
| L-Phenylalanine | **0.5** | 3.03 | 0.015 | 0.09 | Vitamin B12 | 5.00E-06 | 3.70E-06 | 5.00E-06 | 3.69E-06 |
| L-Proline | **2.5** | 21.71 | 0.02 | 0.17 | Inosine | **0.005** | 0.02 | N/A | N/A |
| L-Serine | **1** | 9.52 | 0.03 | 0.29 | Uridine | **0.005** | 0.02 | N/A | N/A |
| L-Threonine | **1** | 8.39 | 0.02 | 0.17 | Urate | **0.005** | 0.03 | N/A | N/A |
| L-Tryptophan | **0.02** | 0.1 | 0.005 | 0.02 | Adenine | **0.01** | 0.07 | N/A | N/A |
| L-Tyrosine • 2Na • 2H2O | **0.5** | 1.9 | 2.88E-02 | 0.11 | Uracil | **0.01** | 0.09 | N/A | N/A |
| L-Valine | **1** | 8.54 | 0.02 | 0.17 | Guanine | **0.01** | 0.07 | N/A | N/A |
| ***D-Glucose*** | 0.2 | 1.11 | 2 | 11.10 | Thymine | **0.005** | 0.04 | N/A | N/A |
| ***Sucrose*** | 0.1 | 0.55 | N/A | N/A | Cytosine | **0.005** | 0.05 | N/A | N/A |
| Calcium Nitrate • 4H2O | 0.1 | 0.42 | 0.1 | 0.42 | Orotate | **0.005** | 0.03 | N/A | N/A |
| Magnesium Sulfate (anhydrous) | **0.05** | 0.42 | 4.88E-02 | 0.41 | ***Spermine*** | **0.145** | 1 | N/A | N/A |
| Potassium Chloride | 0.4 | 5.37 | 0.4 | 5.37 | 3-acetamidopropanal | **0.01** | 0.09 | N/A | N/A |
| Sodium Bicarbonate | 2 | 23.81 | 2 | 23.81 | Catalase | **0.33** | NA | N/A | N/A |
| Sodium Chloride | 2 | 102.67 | 6 | 102.67 | Phenol Red • Na | N/A | N/A | 5.30E-03 | 1.41E-02 |
| Sodium Phosphate Dibasic | 0.8 | 5.64 | 0.8 | 5.64 | Albumax | N/A | N/A | 2.5 | 3.79E-02 |
| HEPES | N/A | N/A | 5.958 | 25 |  |  |  |  |  |
